# Supplementary material for: Are carbon emissions trading and green financial instruments synergistic? -Comprehensive quantitative research based on content analysis
Source: PLoS One. 2024 Mar 7;19(3):e0298601. doi: 10.1371/journal.pone.0298601 (PMC10919601; doi:10.1371/journal.pone.0298601)
Supplement: S1 Data — (ZIP) [file pone.0298601.s001.zip › Data/地级市Z_F工作报告.zip!/╜¡╦╒/╤╬│╟╩╨/└┤╫╘╣1⁄2╓┌║┼í╛┬φ┐╦╩2╛▌═°í┐_2015.docx]

各位代表:

现在,我代表盐城市人民政府向大会作工作报告,请予审议,并请各位政协委员和其他列席人员提出意见。

2014年工作回顾

过去的一年,面对复杂严峻的宏观经济环境和艰巨繁重的改革发展稳定任务,我们深入贯彻党的十八大、十八届三中、四中全会和习近平总书记系列重要讲话精神,坚决落实省委省政府和市委的决策部署,围绕全面建设高水平小康社会奋斗目标、“三先”工作追求、“四城”总体定位和“六大发展”战略布局,积极适应经济发展新常态,坚持稳中求进、改革创新,统筹推进稳增长、调结构、重生态、惠民生、保稳定各项工作,较好地完成了市七届人大三次会议确定的主要目标任务,盐城发生了新的可喜变化。

一、经济发展质效实现新提升。主要经济指标增速位居全省前列。全市实现地区生产总值3836亿元,增长10.9%;一般公共预算收入418亿元,增长14%;固定资产投资2751亿元,增长23.2%;社会消费品零售总额1314亿元,增长13%。全市经济发展总体水平加快向苏北第一迈进。产业转型升级取得重要进展。扎实开展项目推进年、载体建设年活动,优化经济结构,二三产业比重提高0.5个百分点。大力发展“4+3”新兴产业,主营业务收入突破1500亿元。举办环保产业博览会、汽车博览会。东风悦达起亚汽车产销64.6万辆,全市汽车产业销售突破千亿元。成为国家新能源汽车推广应用城市、国家新能源示范城市。实施农业现代化工程,高效设施农业新增面积全省第一,连续三年获评全国粮食生产先进市。成功承办第十六届江苏农业国际合作洽谈会。加快建设31个现代服务业集聚区和100个重点项目,服务业投资增长28.3%。旅游业快速发展,入境游客人数苏北第一。创新驱动战略加快实施。市区“两区两城”标志性创新高地加速崛起,国家知识产权试点城市建设通过验收。积极培育科技型中小企业,建成省级以上研发机构71个,获批国家高新技术企业105家,高新技术产业产值增长17%。建设省级大学科技园。举办第四届沿海发展人才峰会,引进高层次人才创业项目51个。

二、推动改革开放取得新进展。着力推进行政体制改革。进一步简政放权,研究出台市县政府职能转变和机构改革方案,取消调整下放市级行政权力事项294项、行政审批事项153项,审批周期缩短52%。基本建成市县镇村四级便民服务网络。推进市场体制改革。突出全民创业,放开投资领域,推出首批鼓励民间投资“百项千亿”工程,新增各类市场主体7.9万家,居全省前列。制订14家市属重点国有企业改革方案。加快财税金融和价格体制改革,完成1.65万户纳税人“营改增”试点,产生减税效应13.3亿元。贷款增速全省第一。推进农业农村改革。开展土地确权登记市级试点,推广联耕联种面积213万亩,新增市级示范家庭农场100家,新发展农村专业合作组织1071家。推进可持续发展综合改革。《国家可持续发展实验区建设综合配套改革实施方案》获得省委省政府批准。大力发展开放型经济。坚持扩大开放与深化改革互动并进,在全省率先试行外商投资企业登记备案制度。韩资工业园在全省特色产业园区考核中名列第一。新批3000万美元以上利用外资项目30个,进出口总额突破70亿美元。港澳台侨和外事工作取得新的成绩。

三、沿海开发建设呈现新局面。加快重大基础设施建设。盐城港新开工建设万吨级以上泊位8个,大丰港区三期通用码头建成通航,滨海港区10万吨级通用码头开港试航,响水港区一类口岸临时开放获得国家批准。连盐铁路全线开工建设,徐宿淮盐高铁项目取得突破性进展。阜建高速公路、盐城市区至大丰港高速公路快速推进,临海高等级公路全线建成通车,新建国省干线公路102公里。加快重大产业项目建设。列入省沿海开发五年推进计划的78个重点项目,累计完成投资3008亿元。领胜科技等36个投资超10亿元产业项目开工建设,滨海港区2×100万千瓦火电项目获得国家立项。加快载体平台建设。18家重点开发园区在全省整体进位,工业开票销售、工业投资、注册外资实际到账分别占全市的75%、75%、72%,中小企业园项目承载能力进一步增强。市开发区、环保科技城被认定为国家火炬计划特色产业基地,盐城高新区创建国家高新区已报国务院审批,黄海经济区基础设施框架初步拉开,省级滩涂综合开发试验区启动实施。加快沿海合作开发。在海内外成功举办5场有影响力的投资合作洽谈会,引进央企45家。各类合作园区加快建设。

四、统筹城乡发展迈出新步伐。强化中心城市功能。大市区重点城建项目完成投资350亿元。范公路快速路先导段建成通车,新增公交车389辆、出租车300辆,开通市区至大丰城际公交,城市公交客运总量突破1亿人次。倡导低碳环保出行,推广应用新能源汽车1237辆,投放城市公共自行车6000辆。完善城市服务设施,金融城一期基本竣工,新建改造农贸市场49个。实施“美丽夜景靓丽街区”景观亮化工程。强化城市建设管理,创成国家园林城市,国家卫生城市创建工作顺利通过国家综合评审,城市形象和知名度不断提升。积极推进城乡建设。启动实施新型城镇化八大类220项重点工程项目,加快县(市)城、重点中心镇和新型农村社区建设,抓好36个市级城乡统筹试点镇村发展,城镇化率提高到58.2%。改造提升农村公路1127公里,电力、邮政、通信等设施建设取得新的进展。持续改善生态环境。实施十大类130项生态文明建设工程,成为首批国家生态文明先行示范区。推动绿色盐城建设,新增绿化造林14.1万亩,林木覆盖率达25.6%。整治城乡环境,开展城乡河道综合整治行动,强势推进通榆河沿线环境专项整治。实施大气污染防治行动计划,夏秋两季秸秆禁烧实现“零火点”,空气质量全省最好,良好生态成为盐城的环境品牌。

五、人民群众生活有了新改善。推进民生幸福工程八大体系建设,全市财政安排民生支出434亿元,市政府为民兴办20件实事完成年度目标任务。多渠道增加居民收入。新增城镇就业11万人,城镇登记失业率保持较低水平。大力发展镇村经济,开展新一轮扶贫开发,帮扶14.9万低收入口脱贫。城乡居民收入分别增长9.2%、11.6%。发展各项社会事业。推进义务教育基本均衡发展,改善薄弱学校办学条件,新建中小学、幼儿园28所。加强基层医疗卫生、大市区医疗急救体系建设,城乡居民大病保险实现全覆盖。实施文化惠民工程,开展第二届“盐渎风”全民读书月活动,改造提升公共文化场所和革命遗址15个。积极发展新闻出版、广播电视、档案等事业。广泛开展全民健身活动,成功举办沿海湿地国际公路自行车赛。提高社会保障水平。在全国率先推行城乡居民社会养老保险并轨和“四个不出村”便民快捷服务,企业退休人员养老金上调12.8%。在全省率先建立困难群众保障援助和市区困难群众托底救助制度,20多万被征地农民享有基本生活保障。全市整治老旧小区41个,新建各类保障性住房2.5万套。启动实施总投资40亿元的饮用水安全保障十项工程,盐龙湖至城东水厂源水互通工程、城东水厂深度处理工程基本建成。推动城乡区域供水一体化,新保障55.7万农村居民饮用水安全。完成32个农村五保供养服务机构提档升级项目,大市区养老呼叫服务平台投入运营,城市社区居家养老服务中心(站)实现全覆盖。老龄、残疾人、红十字、慈善等事业进一步发展。全力维护社会稳定。深入开展“五大专项行动”,突出抓好信访积案化解、安全隐患整治等工作,实现了信访总量、事故数量和刑事案件发案率“三下降”,司法水平、公众安全感、法治满意度“三上升”。扎实做好食品药品监管、应急管理、消防、民族和宗教事务等工作,连续七年被省评为“平安市”,连续三年被省评为“综治工作先进市”。

推进国防动员、国防后备力量和人民防空建设,加强优抚安置、双拥共建、民兵和预备役工作,军政军民关系进一步密切。

过去的一年,我们深入开展党的群众路线教育实践活动,严格执行中央“八项规定”和省、市委“十项规定”,着力解决“四风”方面存在的突出问题,推动作风建设长效化、常态化。全市性会议数量下降19.3%,以市政府、市政府办公室名义发文数量分别下降47.4%、35.1%,公务接待、公务用车购置、公款出国(境)费用分别下降38.1%、89%和33.8%。强化法治政府建设,主动向市人大及其常委会报告工作,向市政协通报情况,174件人大代表建议、229件政协委员提案全部办结。

各位代表,今天的盐城,发展势头强,城乡变化大,干群状态好,社会风气正,全面小康建设迈出新的坚实步伐。成绩来之不易,这是省委省政府和市委正确领导的结果,是全市人民同心同德、创新创业、共同奋斗的结果。在此,我谨代表市人民政府,向广大干部群众,向人大代表、政协委员,向离退休老同志,向各民主党派、工商联、人民团体和各界人士,向驻盐人民解放军、武警官兵、公安干警、省属驻盐各单位,向所有关心和支持盐城发展的海内外朋友,致以崇高的敬意和衷心的感谢!

各位代表,在肯定成绩的同时,我们也清醒地认识到面临的挑战和问题:经济下行压力加大,部分企业生产经营困难,重大项目和新增长点不多;少数改革措施落地见效不够快,创新驱动能力不强,发展动力还显不足;基本公共服务保障水平不高,城乡居民收入增长不快,农村金融和政府性债务等潜在风险依然存在;政府职能转变还不到位,部门办事效率有待进一步提高。对这些问题,我们一定高度重视,采取有力有效措施切实加以解决。

2015年主要任务

各位代表,当前我市经济社会已进入一个新的发展阶段,我们面临着主动适应新常态、推动发展上台阶的新任务、新要求。盐城发展空间广、潜力大、势头好,各种机遇叠加,潜力和优势正在加速释放,正可谓天时地利人和,具备了迈上新台阶的坚实基础。面对发展仍不充分的市情实际,我们必须牢固坚持发展第一要务不动摇,扎实做好今年各项工作,坚定信心、主动作为,锐意进取、跨越赶超,奋力开创新常态下盐城发展新境界。

今年政府工作的总体要求是:全面贯彻党的十八大和十八届三中、四中全会精神,深入贯彻习近平总书记系列重要讲话精神,按照总书记对江苏工作最新要求,落实省委十二届九次全会和市委六届六次全会总体部署,围绕“建设新盐城、发展上台阶”总定位,主动适应经济发展新常态,牢牢把握稳中求进总基调,坚持以提高经济发展质量和效益为中心,以绿色发展为方向,以国家可持续发展实验区建设为抓手,加快转型发展,深化改革创新,强化生态建设,保障改善民生,确保经济持续健康发展和社会大局和谐稳定,推动盐城经济社会发展迈上新台阶。

全市经济社会发展主要预期目标是:地区生产总值增长10%,一般公共预算收入增长12%,居民消费价格涨幅控制在3%左右,城镇登记失业率控制在4%以内,全社会研发投入占地区生产总值比重2%左右,城乡居民收入分别增长10%和11%,完成节能减排省定目标。

做好今年政府工作,必须讲政治、顺民意、抓大事、办实事。讲政治,就是在政治上、思想上、行动上与以习近平同志为核心的党中央保持高度一致,贯彻落实中央、省委省政府和市委的决策部署,态度坚决、执行有力,确保落地生根。顺民意,就是把盐城人民对美好生活的向往作为奋斗目标,想问题、办事情、做决策必须符合实际、顺应民意、赢得民心。抓大事,就是着眼长远,全力突破一批事关全局的重大基础设施、民生工程和产业项目,常抓不懈、久久为功。办实事,就是像抓经济建设一样抓民生保障,全力办好各项民生实事,努力让人民群众有更好的教育、更稳定的工作、更满意的收入、更可靠的社会保障、更高水平的医疗卫生服务、更舒适的居住条件、更优美的环境。

今年重点做好以下八项工作:

一、更加突出质量效益,促进经济稳健增长。坚持综合施策、多措并举,稳定经济运行,挖掘增长潜力,推动全民创业,促进经济稳中有进、行稳致远。

力促现有企业提质增效。加强经济运行调节,推动生产要素、政策资源、政府服务向实体经济集聚。继续实施大企业(集团)培育工程,开展亏损企业扭亏脱困三年行动计划,力争企业增长面在60%以上。用足用好支持企业发展若干政策专项和中小微企业应急过桥担保资金,帮助企业争取国家和省更多专项扶持。促进金融机构向上争取信贷规模,拓展融资渠道,力争新增社会融资规模650亿元。落实结构性减税和降费政策,规范涉企收费,减轻企业负担。科学配置用地计划,确保大项目、好项目用地。

增强投资和消费拉动力。深化投融资体制改革,放宽市场准入,积极推行政府与社会资本合作(PPP)等多种投融资模式。组织实施“三重”、“八大类”、工业“三百”等重点工程项目,扎实开展“五抓五比”活动,抓洽谈签约、比协议投资额,抓注册到账、比资金到位数,抓开工建设、比实物投入量,抓投产达效、比新增长点,抓软环境建设、比服务效能,新增100个以上销售超亿元的新增长点,固定资产投资突破3000亿元,增长18%。大力促进消费升级,加快国家信息消费试点城市建设,积极培育养老、健康、文化、旅游等消费热点,合理引导房地产消费需求,社会消费品零售总额增长13%。

大力发展镇村经济。以发动全民创业为路径,强化规划引领、政策支持和考核激励,催生“铺天盖地”的新增长点,厚实全市经济基础。制定完善鼓励镇村经济发展的政策措施,实施民间投资“百项千亿”工程,搭建全民创业十大服务平台,支持农民、大学生自主创业和外出务工人员返乡创业,让一切创造财富的源泉充分涌流。新登记企业2.2万户,民营经济增加值占比提高到70%。加快建设中小企业创业园、农民创业园,开展百家创业园区优化升级活动,新建标准厂房200万平方米。坚持因地制宜,宜工则工、宜农则农、宜商则商、宜游则游,在严守环保安全底线的基础上,大力发展劳动密集型产业、农产品加工流通业、生态旅游业和家政服务业,扶持经济薄弱镇村发展,90%以上的村集体经济收入超过15万元。

二、深化产业结构调整,推动经济转型发展。坚持把转方式调结构放在突出位置,开展“项目载体突破年”活动,大力发展新产业、新业态、新模式,构建绿色产业体系,推动产业结构向中高端攀升,树立新产业经济标杆。

做大做强新兴产业。以建设国家战略性新兴产业集聚发展试点城市为契机,大力实施“4+3”新兴产业培育计划,推进20个专业特色园区、100个重点项目建设,培植一批竞争力强的行业龙头企业,新兴产业销售收入增长30%以上。大力发展千万千瓦风电、千兆瓦级光伏等可再生能源产业,加快建设国家新能源示范基地,清洁能源发电占全市用电的20%。大力发展节能环保产业,加快建设省环保产业基地、省大气污染防治产业创新基地,着力打造国内领先的环保科技城。

改造提升传统产业。开展工业转型升级十大行动,推动汽车、机械制造、纺织、化工等支柱产业提档升级,提升产业竞争力。深入实施“千百十”工程,完成千万元以上技改项目500项,技改投资增长25%以上。积极发展智能制造、绿色制造,市域信息化发展水平总指数提高到80以上。实施东风悦达起亚汽车第三工厂15万辆乘用车技改项目,推进三菱扶桑商务客车项目、中车集团轨道客车项目尽快实施,奥新公司15万辆轻量化电动车一期项目建成投产,为实现汽车整车、零部件和汽车服务业“三个千亿”目标打好基础。推进建筑产业现代化,鼓励建筑企业资质升级,建筑业产值达到1550亿元。

集聚发展现代服务业。深入实施服务业集聚区提升工程,加快发展现代物流、科技金融、会计审计等生产性服务业,推进文化创意、研发设计等新兴产业与传统产业融合发展,做大做强百家服务业龙头企业。成立市级金融控股公司,着手组建地方法人银行。加快发展电子商务,培育基于网络的数字化制造、内容服务、平台经济等新业态,推进大数据、电商快递、软件和服务外包产业园区建设。加快发展旅游业,建设旅游精品线路、精品工程,推进丹顶鹤保护区、麋鹿保护区、大洋湾城市公园等重点项目建设,积极创建国家森林公园和湿地公园,做好沿海湿地核心区申报自然遗产前期工作,规划建设沿海湿地旅游经济带。

增强创新驱动力。积极构建以企业为主体、产业为方向、市场为导向、人才为支撑的创新体系,推进产学研一体化,建设国家创新型城市。加快建设国家高新区、智慧科技城、大学科技园和各类创新创业“孵化器”。大力实施企业“三自主”创新工程,鼓励企业设立自主研发机构、申报自主知识产权、创立自主品牌,力争大中型企业市级以上研发机构全覆盖,专利授权量增长10%。发挥创新创业引导基金作用,培育10家创新型领军企业、100家高新技术企业、1000家科技型中小企业。积极发展人力资源服务业,依托科技部国家领军人才创新驱动中心和中国科协盐城海智工作基地,引进高层次创新创业团队和专业人才。办好第五届沿海发展人才峰会。完善人才流动、培养、使用、激励机制,营造人尽其才、才尽其用的良好环境。

三、转变农业发展方式,提升农业现代化水平。大力发展绿色农业,打响绿色品牌,建设国家现代农业示范基地,为全省农业迈上新台阶带好头、领好向。

构建现代农业产业体系。深入推进农业结构战略性调整,加快发展现代园艺业、特色水产业和休闲观光农业,重点打造百万亩沿海现代渔业示范带和国省道沿线现代农业示范带,提升现代农业园区建设水平,新增高效设施农(渔)业面积20万亩。推进农产品加工集中区建设,招引一批农产品精深加工项目,重点培植中粮肉食、辉山乳业、银宝渔业、华大水产等100家农业龙头企业,加工业产值突破2000亿元。完善农产品市场体系,推进联产联销,鼓励出口创汇,推动农业“接二(产)连三(产)”发展,提高农产品附加值,促进农民持续增收。扩大农业对外合作,打造海峡两岸现代农业合作示范基地。

培育新型农业经营主体。积极培育专业大户、家庭农场、农民合作社、农业企业,新型农业经营主体适度规模经营占比提高5个百分点,农村专业合作组织入社农户突破150万户。推进农村土地经营权有序流转,发展多种形式的适度规模经营,推广联耕联种面积300万亩,并向联管联营拓展。开展农民职业技能培训2.5万人次,建设一支有文化、懂技术、会经营的职业农民队伍。

加强农业质量品牌建设。开展耕地质量保护和提升行动,发展综合循环农业,强化农业面源污染综合整治,推进生态农业示范基地建设。健全农产品标准化生产、投入品监管、安全可追溯、质量诚信、安全监管和检测“六大体系”,确保“舌尖上的安全”。建立产地准出示范基地100个,“三品”产量占比达到45%。制定农业品牌发展规划,整合打造10个竞争力强的区域公共品牌。

提升农业装备和技术水平。加强农业基础设施建设,全面兴修水利,推进黄河故道综合开发,大规模建设高标准农田。加快农业装备产业园建设,推广各类大中型农机具3.5万台(套),主要农作物综合机械化水平达到85%。强化农业科技创新,健全农技推广服务体系,农业科技贡献率提高3个百分点。积极发展农业电商平台,新建村级信息服务站500个以上,创建全国农业信息化示范市。

四、推进“三港”联动发展,打造沿海开发升级版。积极融入“一带一路”国家战略,深入实施省沿海开发六大行动计划,加快沿海绿色开发,实现沿海中部崛起。

推动港口功能升级。理顺盐城港管理体制,扩大港口吞吐量,力争跻身亿吨大港。提升大丰港区发展定位,优化港口规划,全面展开15万吨级通用码头、集装箱码头、邮轮码头等四期工程前期工作。推进滨海港区中电投煤炭码头和南区液体散货码头建设,北区通用码头投入运营。开工建设响水港区2个5万吨级、射阳港区2个3.5万吨级码头。加快四个港区深水航道建设,推进滨海港区疏港航道、大运河至通榆河船闸改造工程,开展淮河入海水道航道工程前期工作,完善港口集疏运体系,提升河海联运能力。鼓励有条件的港区申报一类开放口岸。

推动临港产业升级。坚持高轻新、基地化发展方向,加快实施列入省、市沿海开发计划的重大项目,开工建设滨海港区2×100万千瓦火电、三峡新能源海工装备等项目,推进赛欧帕克干细胞、明月海洋生物等项目,发展壮大绿色石化、清洁能源、新型材料、海洋生物等产业。开发建设临海高等级公路经济带,推动形成支撑沿海新一轮发展的绿色经济走廊。强化沿海战略资源保护和综合开发利用,大力推广绿色开发模式。成立市级能源投资发展公司,合作开发沿海风光资源。

推动载体平台升级。坚持产城融合发展,实施临海城镇培育行动计划,试行“区镇合一”体制,做优沿海城市形态,打造精品港口城镇,完善功能配套,为产业发展和人气集聚提供支撑。提升港区重大项目承载功能,支持有条件的沿海经济区争创国家级、省级开发区。加快滩涂综合开发试验区、沪苏(大丰)产业联动集聚区和各类合作园区建设。

五、增强中心城市功能,扎实推进新型城镇化。着眼以人为本、惠民利民,以低碳生态环保理念,推进城市转型发展和城乡统筹发展。

建设现代化中心城市。进一步优化大市区空间布局,推进“南移东扩”,组织实施十类百项城建重点项目,完成投资208亿元,切实增强中心城市辐射带动能力。完善城市路网,全面开工建设城市内环高架快速路系统,年内完成投资25亿元,实施世纪大道东延、东进路西延、鹿鸣路改造等道路工程。新增一批新能源出租车、公交车和公共自行车,更加方便市民低碳出行。强化公共配套,建成美术馆,改造城市规划展览馆,建设提升聚龙湖商务中心、跃进河商业街区。规划建设城市文化娱乐中心、体育休闲中心和市博物馆,突出抓好国际化社区、学校和医院建设,提高城市国际化水平。加强智慧城市网络基础设施建设,主要公共区域实现免费WiFi(无线网络)全覆盖。做美城市环境,推进老旧小区整治、城中村和棚户区改造,完成投资45亿元。提高住宅小区物业管理覆盖率。巩固国家卫生城市创建成果,积极创建省优秀管理城市和全国文明城市。

推进城镇化健康发展。坚持规划引领、示范带动,优化城镇化布局,完成专项规划编制任务,推进“多规合一”,加快城乡基础设施、公共服务等“六个一体化”,城镇化率达到60%。提高县(市)城建设管理水平,打造一批特色鲜明的工业强镇、商贸重镇、旅游名镇和卫星城镇,促进农村人口就地城镇化。突出抓好13个全国重点镇、30个市级重点中心镇、200个新型农村社区和3000个规划村庄建设,36个统筹城乡发展试点镇村全部达到市级示范标准。

突破重大交通基础设施建设。抢抓国家铁路新一轮建设和规划修编的重大机遇,全力争取连接大上海和杭州湾的“盐通苏嘉”高铁列入国家专项规划,盐城往南京方向的高铁连接线列入省专项规划,扎实做好项目前期工作。开工建设盐城至淮安往北京方向的高铁,加快建设连盐铁路和沿海铁路大通道,缩短盐城到上海等中心城市的时空距离,推动盐城县县通高铁,早日迈向“高铁时代”。

抓紧编制大市区连接“一港四区”的快速铁路网中长期规划,筹建市铁路投资发展基金。开工建设南洋机场新航站楼,加快完成跑道延伸工程,加密优化航线航班,大幅提高空港客货运能力。做好宁盐高速公路项目前期工程,推进省道331、233等干线公路建设,阜建高速公路建成通车,盐城市区至大丰港高速公路基本建成。改造农村公路500公里、桥梁160座,镇村公交开通率达35%,进一步改善农村居民出行条件。

六、加强生态文明建设,彰显绿色发展特色。以绿色发展倒逼结构调整、践行生态文明、提升城乡品质,引领盐城全面走向绿色发展新时代。

大力发展绿色经济。开展国家生态文明先行示范区建设,发展绿色能源、节能环保、生态休闲旅游和绿色农业,推动绿色增长。推进30项重点节能技改工程和150项重点减排工程,加快淘汰落后工艺、落后产能,强化节能减排年度考核。按照新环保法和新安全生产法的要求,开展化工园区安全环保专项整治,推进绿色转型。开工建设市区静脉产业园。建成省生态市和国家环保模范城市,省级以上重点园区全部建成省级生态园区。全市绿色发展综合指数提高到70.7。

强化城乡环境治理。继续开展“931”城市环境综合整治行动,实施城郊结合部、背街小巷、建筑工地整治等158个项目。全面完成5930个村庄环境整治任务,确保通过省级考核验收。深入实施“碧水蓝天”工程,着力解决群众反映强烈的突出问题。推进城乡河道综合整治三年行动计划,疏浚整治市区核心区河道38条、农村大中小沟1.2万条,完成投资15亿元。加大大气污染防治力度,整治燃煤锅炉450个,加强建筑扬尘治理、秸秆禁烧和综合利用工作,保持空气质量全省第一。

建设绿色美丽家园。实施全市绿化造林十大工程、市区园林绿化十大工程,提升公园绿地景观效果,创成全国绿化模范城市。积极申办省第十届园艺博览会,启动国家生态园林城市创建工作,参加国际花园城市竞赛活动。加快沿海五个万亩林场建设,完善生态防护林体系。开展绿色村庄达标创建活动,建设高标准农田林网。全市新增成片造林10万亩,新抚育森林15万亩,新增城市绿地760公顷,林木覆盖率达到26%,让盐城水更净、天更蓝、地更绿、环境更优美。

七、全面深化改革开放,增强发展动力和活力。坚持问题导向,更大力度破除制约发展的体制机制障碍,激发改革推动力,增创开放新优势。

突破重点领域改革。加快实施国家可持续发展实验区建设综合配套改革,制定实施计划,狠抓落地见效,着力构建产业优化升级、资源科学开发、生态环境保护、城乡一体发展、社会治理创新五大机制,积极争取建立实验区建设部省联席会议制度。完成市县政府机构改革和公车改革任务,稳步推进不动产统一登记制度改革。深化国有企业改革,推动市级投融资平台向实体化、市场化方向转型。扩大“营改增”范围,开展小微企业转贷方式创新试点,启动农村土地承包经营权抵质押融资试点。严格执行新预算法,推行预决算公开,加强财政资金绩效审计,规范政府性债务管理。全面推进农村土地承包经营权确权登记颁证,扩大产权交易平台试点。推进海域使用权制度改革。尊重基层首创精神,鼓励支持各地结合实际,大胆探索,大胆实践。

建设园区开放高地。坚持低碳绿色循环发展,调整完善园区发展规划,从体制机制创新、人才队伍建设、园区环境提升入手,促进各级各类园区转型升级,加快建设开放型经济高地。抢抓中韩自贸区建设机遇,推进中韩(盐城)产业园建设,全力推动形成两国政府(商务部门)主导、地方与企业参与、市场化建设的开发机制,打造国际合作创新园和生态园。深化两岸两地产业合作,打造台资产业集聚和创新发展高地。积极推动黄海经济区与市开发区深度融合,借助国家级开发区平台优势,加快盐田开发和沿海园区建设,打造转型升级、绿色发展示范区。发挥综合保税区平台作用,争取一批跨国公司在我市建设生产制造和物流基地。强化全天候招商、产业链招商和专业化招商,重点面向世界500强企业、央企、大型民企,精心组织“春夏秋冬”四季招商战役,凝心聚力抓项目、锲而不舍抓项目、殚精竭虑抓项目、攻坚克难抓项目,力争突破更多大项目、好项目。扩大外贸出口,发展跨境贸易,进出口总额增长10%。实施“走出去”战略,加快企业国际化步伐。完善激励约束机制,加大对开发园区考核力度,促进进位争先。

八、着力保障改善民生,维护社会和谐稳定。深入实施民生幸福工程,像落实发展指标一样,落实民生任务。今年市政府将继续为民兴办20件实事,完成投资88.5亿元。全市公共财政支出73%以上用于民生保障,其中市级财政预算安排民生支出46亿元,新增财力82%用于民生改善。

更加注重保障基本民生。突出解决饮用水安全问题,抓紧做好引进清洁水源专项规划和向上争取工作,推进饮用水安全保障十大工程,完成投资23亿元。抓好通榆河、蟒蛇河、射阳河、串场河等清水廊道建设,开工建设盐龙湖水厂一期工程、通榆河取水口原水预处理净化工程,实现城乡区域供水全覆盖。实施更加积极的就业政策,新增城镇就业8万人以上。深化收入分配制度改革,多渠道增加城乡居民工资性收入、经营性收入和财产性收入,继续提高企业退休人员基本养老金和城乡居民基础养老金,逐步提高城乡居民医保财政补助标准,合理提高机关事业单位工作人员收入水平。深入推进精准扶贫、片区开发,确保低收入贫困户全部脱贫。提升社会保障水平,启动实施机关事业单位工作人员养老保险制度改革,城乡居民基本养老、基本医疗、失业保险参保率稳定在96%以上,落实被征地农民基本生活保障制度,实现困难群众保障援助和托底救助制度全覆盖。扩大住房公积金制度覆盖面。新建各类保障性住房2.3万套。加快建设市社会福利院二期工程,鼓励社会力量进入养老体系建设,大市区485个社区(村)全部建有居家养老服务中心(站),全市所有农村敬老院达到省定标准。

繁荣发展各项社会事业。培育和践行社会主义核心价值观,弘扬铁军精神和创业精神,全面提升市民素质,建设社会文明程度高的厚德盐城。继续实施文化惠民工程,加大“书香盐城”建设力度,打造城市“15分钟文化圈”、农村“10里文化圈”,万人拥有公共文化服务设施面积1300平方米以上。实施“五个一”文化亮点工程,推出一台综合性大戏,出版一套宣传丛书,改造一批重点文化场馆和革命遗址,规划建设一个标志性的现代文化综合体,引进一批文化重点企业单位,支持首批20家市级文化产业示范基地建设,推动文化建设迈上新台阶。

全面深化医药卫生体制改革,加快建设现代医疗卫生体系。采取政府发债贴息措施,重点加强50个乡镇卫生院和社区卫生服务中心、456个村卫生室和社区卫生服务站标准化建设,培训基层医疗卫生人员1.7万人次,提高基层医疗服务水平。建立疾病应急救助专项基金,重视解决因病致贫、因病返贫问题。全面推进教育现代化工程,实施新一轮教育布局调整,突出办好农村教育,促进城乡教育协调发展。启动市区串场河小学、城南高中、环保职业学院等项目建设,推进高等师范学校整合提升,积极做好盐城大学筹建工作。加快发展体育事业,举办市第七届运动会。关心支持老干部、关工委工作,发展妇女儿童事业,加强防震减灾、气象、援疆、援青等工作。

大力推进社会治理创新。以法治盐城建设为引领,深化平安盐城建设,健全立体化现代化社会治安防控体系,保障公共安全。强化基层基础工作,推进村务公开,创建示范社区,推行“政社互动”社会治理模式,发挥行业协会等社会组织在社会治理中的作用。完善重大决策社会稳定风险评估机制,积极推进“阳光信访”,着力化解信访积案、镇村债务、农村金融风险,确保社会大局稳定。加强食品药品安全监管。严格落实安全生产主体责任和监管责任,坚决遏制重特大安全事故发生。健全应急管理机制,提高突发事件应急处置能力。

深入开展国防教育,加强国防动员、国防后备力量和人民防空建设,推进军民融合深度发展,争创全国双拥模范城“五连冠”。

各位代表,编制“十三五”发展规划意义重大,也是今年的一项重要任务。我们将科学谋划“十三五”发展大局,加强对重大项目和重大政策的研究,使规划更加符合市情实际,更加顺应群众期盼。同时,积极向上对接,争取一批事关全局的重大项目列入国家和省“十三五”规划,为盐城未来发展奠定坚实基础。

加强政府自身建设

今年政府工作任务重、要求高、压力大,加强自身建设尤为重要。适应新常态,关键在状态。我们将深入开展“作风建设深化年”活动,以更好的精神状态、更强的担当意识、更实的工作作风,努力开创政府工作新局面,建设人民满意的法治政府、廉洁政府和服务型政府。

一、坚持依法行政,建设法治政府。把政府工作全面纳入法治轨道,依法行使权力履行职责,做到法定职责必须为、法无授权不可为,坚决纠正不作为、乱作为。坚持依法决策,建立重大决策合法性审查、终身责任追究及责任倒查机制。坚持严格规范公正文明执法,深化行政执法体制改革,积极推进综合执法,加大关系群众切身利益的重点领域执法力度。进一步深化政务公开,自觉接受党内监督、人大法律监督、政协民主监督、司法监督、审计监督,重视人民群众监督和社会舆论监督,认真办理人大代表建议、政协委员提案,切实改进政府工作。政府全体工作人员都要增强法治观念,提高依法行政能力,做学法知法守法的表率。

二、坚持从严治政,建设廉洁政府。严格执行中央“八项规定”和省、市委“十项规定”,深化“四风”整治,巩固和拓展党的群众路线教育实践活动成果,打好作风建设持久战。认真落实政府系统党风廉政建设责任制和责任追究制,强化领导干部“一岗双责”,管好自己、带好队伍、树好形象。健全廉政风险防控制度,加强对政府内部权力制约,强化内部流程管控,防止权力滥用。坚持以零容忍态度惩治腐败,严肃查办发生在重点领域、关键环节和群众身边的腐败案件,做到有腐必反、有贪必惩。大力倡导艰苦奋斗、厉行节约,带头过紧日子,压降“三公”经费,勤俭办一切事业。加强廉政文化建设和反腐倡廉教育,努力建设信念坚定、作风正派、清正廉洁的高素质公务员队伍。

三、坚持勤政为民,建设服务型政府。牢记为民服务宗旨,强化政府公共服务职责,加强基本公共服务网络建设,让城乡居民就近获得基本公共服务。创新公共服务方式,推行政府购买服务制度。大力推进电子政务、网上办公,提高服务效能,构建综合高效的政务服务平台。深化行政审批制度改革,打造全省盖章最少、收费最少、材料最少和流程最短“三少一短”政务服务环境。健全领导干部联系群众、服务企业、挂钩村居等制度,解决联系服务群众“最后一公里”问题。始终把干事创业谋发展作为最大责任,敢于担当,甘于奉献,坚决纠正懒政、怠政和为官不为现象。以踏石留印、抓铁有痕的劲头,抓推进、抓落实、抓突破,坚决不做花花哨哨的事,坚决不做急功近利的事,坚决不做劳民伤财的事,努力创造出经得起实践、人民和历史检验的业绩。

各位代表!盐城正站在一个新的历史起点上,全面完成今年各项目标任务,责任重大,使命光荣。让我们紧密团结在以习近平同志为核心的党中央周围,在省委省政府和中共盐城市委的坚强领导下,紧紧依靠全市人民,解放思想、开拓奋进,凝心聚力、攻坚克难,全力推动经济社会发展迈上新台阶,为建设创业开放生态幸福的美丽盐城而努力奋斗!
